# The effects of acute high-intensity aerobic exercise on cognitive performance: A structured narrative review

**DOI:** 10.3389/fnbeh.2022.957677

**Published:** 2022-09-23

**Authors:** Mizuki Sudo, Joseph T. Costello, Terry McMorris, Soichi Ando

**Affiliations:** ^1^Physical Fitness Research Institute, Meiji Yasuda Life Foundation of Health and Welfare, Tokyo, Japan; ^2^Extreme Environments Laboratory, School of Sport, Health and Exercise Science, University of Portsmouth, Portsmouth, United Kingdom; ^3^Institute of Sport, University of Chichester, Chichester, United Kingdom; ^4^Graduate School of Informatics and Engineering, The University of Electro-Communications, Chofu, Japan

**Keywords:** cognition, dual task, cerebral blood flow, cerebral oxygenation, cerebral metabolism, neuromodulation

## Abstract

It is well established that acute moderate-intensity exercise improves cognitive performance. However, the effects of acute high-intensity aerobic exercise on cognitive performance have not been well characterized. In this review, we summarize the literature investigating the exercise-cognition interaction, especially focusing on high-intensity aerobic exercise. We discuss methodological and physiological factors that potentially mediate cognitive performance in response to high-intensity exercise. We propose that the effects of high-intensity exercise on cognitive performance are primarily affected by the timing of cognitive task (during vs. after exercise, and the time delay after exercise). In particular, cognitive performance is more likely to be impaired during high-intensity exercise when both cognitive and physiological demands are high and completed simultaneously (i.e., the dual-task paradigm). The effects may also be affected by the type of cognitive task, physical fitness, exercise mode/duration, and age. Second, we suggest that interactions between changes in regional cerebral blood flow (CBF), cerebral oxygenation, cerebral metabolism, neuromodulation by neurotransmitters/neurotrophic factors, and a variety of psychological factors are promising candidates that determine cognitive performance in response to acute high-intensity exercise. The present review has implications for recreational, sporting, and occupational activities where high cognitive and physiological demands are required to be completed concurrently.

## Introduction

A growing body of evidence suggests that acute moderate-intensity exercise improves cognitive performance (Lambourne and Tomporowski, 2010; Chang et al., 2012; Ando et al., 2020; McMorris, 2021). It has been speculated that the relationship between exercise intensity and cognitive performance is inverted-U shaped (Lambourne and Tomporowski, 2010; Chang et al., 2012; McMorris, 2021). In the inverted-U theory, acute exercise gradually increases arousal to an optimal level from rest to moderate intensity and thus improves cognitive performance. A recent review summarized that improvements in cognitive performance following moderate-intensity exercise are frequently accompanied by the changes in brain activation assessed by electroencephalogram (EEG) (Kao et al., 2020), which appears to support the theory that acute exercise alters brain activity and that this is associated with cognitive performance. Acute high-intensity aerobic exercise leads to metabolic, circulatory, and neurohormonal changes at the level of the brain (Ide and Secher, 2000; Meeusen et al., 2001; Nybo and Secher, 2004; Ogoh and Ainslie, 2009; Seifert and Secher, 2011). In contrast to moderate-intensity exercise, theoretically, high-intensity exercise may, therefore, also lead to altered, and potentially impaired cognitive performance. Indeed, the inverted-U theory predicts that high-intensity exercise increases arousal levels beyond the optimal level and leads to a temporary reduction in cognitive performance. However, the current literature base detailing the effects of high-intensity exercise on cognitive performance is not fully supportive of this theory and is somewhat ambiguous and contradictory (Browne et al., 2017; Moreau and Chou, 2019; Cantelon and Giles, 2021; McMorris, 2021; Zheng et al., 2021).

Dietrich and Audiffren (2011) proposed the hypofrontality hypothesis to explain how acute high-intensity exercise affects cognitive performance. The prefrontal cortex (PFC) orchestrates higher-order brain function including cognitive function (Miller and Cohen, 2001; Cools and Arnsten, 2021) and is thought to play a central role in cognitive performance. Acute exercise activates brain regions including motor and sensory cortices, insular cortex, and cerebellum (Williamson et al., 1997; Christensen et al., 2000; Hiura et al., 2014). Hence, the hypofrontality theory speculates that extensive activation of motor and sensory systems during high-intensity exercise likely attenuates higher-order functions of the PFC as the brain has finite metabolic resources (Dietrich and Audiffren, 2011). More recently, McMorris proposed an interoceptive model to explain the effects of high-intensity exercise on cognitive performance (McMorris, 2021). This model offers a more holistic overview of the interaction as it incorporates motivation, perceived effort costs, and perceived availability of resources, together with regional activations and neurotransmitter releases in the brain. Nevertheless, to date, the physiological and psychological mechanism(s) mediating the effects of acute high-intensity exercise on cognitive performance are poorly understood.

In this review, we first summarize the findings of studies investigating the exercise-cognition interaction, especially focusing on high-intensity aerobic exercise. Then, we explore methodological and physiological factors which may alter cognitive performance in response to high-intensity exercise. This review has implications for recreational, sporting, and occupational activities where high cognitive and physiological demands are simultaneously required.

## Methodology

A literature search was undertaken using Pubmed to identify studies that examined the effects of high-intensity aerobic exercise on cognitive performance, assessed during and/or after exercise. The reference lists of relevant articles were also searched. The searches were undertaken in February 2022 and relevant articles were obtained. This review focused on healthy adults, and no restrictions were placed on publication date, study design, methodology, or method of assessing cognitive performance. High-intensity aerobic exercise was defined as exercise equating to ≥ 80% maximum power output (Browne et al., 2017), ≥ 80% maximal oxygen uptake ($\dot{\text{V}}\,{\text{O}}_{2}$) (McMorris, 2016b), or equivalent [e.g., ≥ 80% maximal heart rate (HR)]. Physiological demands are different between continuous and intermittent high-intensity exercise. Thus, we included high-intensity aerobic exercise in this review, and studies incorporating high-intensity interval exercise (HIIE) were considered outside the scope of this review. If specifically interested in this, readers are referred to a recent review that has already explored the effects of HIIE on executive function (Hsieh et al., 2021). In addition, we did not include studies conducted in extreme environments, such as hypoxia and hot/cold environments. However, we referred to evidence from HIIE studies, or studies in extreme environments, for discussion since physiological mechanisms underlying cognitive improvement/impairment in response to HIIE exercise or exercise in extreme environments are, at least partly, shared with those induced by high-intensity aerobic exercise.

## Results

Details of the included studies are shown in Table 1, comprising a total of 40 studies (assessed during exercise only, *n* = 20; assessed both during and after exercise, *n* = 3; assessed after exercise only, *n* = 17). In many studies, cognitive performance was impaired during high-intensity exercise (Chmura et al., 1994; McMorris and Keen, 1994; Brisswalter et al., 1997; McMorris et al., 2009; Labelle et al., 2013; Wang et al., 2013; Dutke et al., 2014; Mekari et al., 2015; Schmit et al., 2015; Smith et al., 2016; Gonzalez-Fernandez et al., 2017; Tempest et al., 2017; Komiyama et al., 2020; Stone et al., 2020). In these studies, impairments in both reaction time (RT) and accuracy were frequently observed. Seven studies reported no changes in cognitive performance (Travlos and Marisi, 1995; Fery et al., 1997; Ando et al., 2011; Dutke et al., 2014; Ciria et al., 2019; Tempest and Reiss, 2019; Komiyama et al., 2020). Five studies reported improvement in RT and/or accuracy during high-intensity exercise (McMorris and Graydon, 1997; Huertas et al., 2011; Shields et al., 2011; Davranche et al., 2015; Tempest et al., 2017).

**TABLE 1:** Summary of the findings.

References	Participants (F)	Category of cognitive task	Cognitive task(s) (number of trials, duration)	Exercise modality/intensity	Exercise duration (high intensity only) or until exhaustion	Timing of cognitive task	Physiological variables	Main findings
Chmura et al. (1994)	*N* = 22 (0) Young	Psychomotor task	Choice RT (30 trials, 107 s)	Cycling Near maximal (300 W)	6 min	During	Blood catecholamine, lactate	RT: impairment
McMorris and Keen (1994)	*N* = 12 (4) Young	Psychomotor task	Simple RT (15 trials)	Cycling 100% maximum workload	Not reported	During	-	RT: impairment
McMorris and Graydon (1997)	*N* = 12 (0) Young	Attentional task executive function	Soccer specific visual search task (30 slides) Soccer specific decision making task (15 trials)	Cycling 100% maximum power output	Not reported	During	-	Visual search: improvement Decision making: improvement
McMorris et al. (2009)	*N* = 24 (0) Young	Executive function	Flanker task (96 trials)	Cycling 80% maximum aerobic power	15 min or until exhaustion	During	Blood catecholamine, adrenocorticotropic hormone, cortisol	RT: impairment Accuracy: impairment
Ando et al. (2011)	*N* = 12 (0) Young	Executive function	Flanker task (40 trials, 3 min 20 s)	Cycling 80% $\dot{\text{V}}\,{\text{O}}_{2}$*_peak_*	6.5 min	During	Cerebral oxygenation	EMG-RT≈ Accuracy≈
Huertas et al. (2011)	*N* = 18 (0) Young	Executive function	Attention network test (modified flanker task, 480 trials, 25 min)	Cycling 95% LT	25 min	During	Blood lactate	RT: improvement
Shields et al. (2011)	*N* = 30 (15) Young	Attentional task	Visual threat-detection task (256 trials)	Cycling 80% maximal HR	Not reported	During	-	RT: improvement Accuracy: improvement
Labelle et al. (2013)	*N* = 37 (18) Young High fit = 16 Low fit = 21	Executive function	Stroop task (80 trials)	Cycling 80% peak power output	6.5 min	During	-	Accuracy: impairment RT variability: impairment in lower fit
Wang et al. (2013)	*N* = 80 (31) Young	Executive function	Wisconsin card sorting test (128 response cards)	Cycling 80% HRR	30 min	During	-	Performance: impairment
Dutke et al. (2014)	*N* = 60 (14) Young	Memory task attentional task (simultaneously)	Word comparison (Primary task, 60 trials, 27 min) Interval production (Secondary task, press a button every 2 s, 27 min)	Cycling 120% AT	> 27 min	During	-	Number of correct response≈ Response time≈ Interval production error: impairment
Davranche et al. (2015)	*N* = 14 (3) Young	Executive function	Simon task (200 trials, 4 min)	Cycling 20% above VT	20 min	During	-	RT: improvement Accuracy≈
Mekari et al. (2015)	*N* = 19 (12) Young	Executive function	Stoop task (30 trials × 2 blocks)	Cycling 85% peak power output	9 min	During	Cerebral oxygenation	RT: impairment Accuracy: impairment
Schmit et al. (2015)	*N* = 15 (5) Young	Executive function	Flanker task (40 trials, as many blocks as possible)	Cycling 85% maximal aerobic power	Until exhaustion	During	Cerebral oxygenation	RT≈ Accuracy: impairment
Smith et al. (2016)	*N* = 15 (9) Young	Executive function	Go/No-Go task (100 trials, 2 min)	Running 90% HRR	10 min	During	-	RT: impairment Accuracy: impairment
Gonzalez-Fernandez et al. (2017)	*N* = 24 (12) Young	Psychomotor task	Psychomotor vigilance task (mean 46.8 trials)	Cycling 100% ventilatory anaerobic threshold	5 min	During	-	RT: impairment
Tempest et al. (2017)	*N* = 14 (5) Young	Executive function	Flanker task (2 min × 10 blocks) 2-back task (60 trials × 10 blocks, 20 min)	Cycling 10% above VT	60 min	During	Cerebral oxygenation	RT (flanker task): improvement Accuracy (n-back): impairment
Ciria et al. (2019)	*N* = 20 (0) Young	Attentional task	Oddball task (20 min)	Cycling 80% $\dot{\text{V}}\,{\text{O}}_{2}$*_peak_*	20 min	During	EEG	RT≈ Accuracy≈
Tempest and Reiss (2019)	*N* = 13 (7) Young	Executive function	N-back task (0-back, 60 trials; 2-back, 60 trials)	Cycling 115% first ventilatory threshold (VT1)	16 min	During	Cerebral oxygenation (fNIRS)	Response time≈ Accuracy≈
Komiyama et al. (2020)	*N* = 17 (0) Young	Executive function	Spatial delayed response Go/No-Go tasks (24 trials, ∼5 min)	Cycling 80% $\dot{\text{V}}\,{\text{O}}_{2}$*_peak_*	8 min	During	Middle cerebral artery blood velocity Cerebral oxygenation	RT≈ Accuracy: impairment
Stone et al. (2020)	*N* = 13 (5) Young	Executive function	Cedar operator workload assessment tool	Running 100% HRR	Until exhaustion	During	Cerebral oxygenation	Accuracy: impairment
Travlos and Marisi (1995)	*N* = 20 (0) Young High fit = 10 Low fit = 10	Attentional task psychomotor task	Random number generation test Choice RT (15 trials × 4)	Cycling during 80% $\dot{\text{V}}\,{\text{O}}_{2}$*_max_* and after volitional exhaustion	Until exhaustion (> 10 min)	During/immediately after	-	Random number generation test (during)≈ RT (after)≈
Brisswalter et al. (1997)	*N* = 20 (0) Young High fit = 10 Low fit = 10	Psychomotor task	Simple detection RT (20 trials)	Cycling during 80% maximal aerobic power	10 min	During/1 min after	-	RT (during): impairment in only low fit Accuracy≈ in both groups
Fery et al. (1997)	*N* = 13 (0) Young	Memory task	Short-term memory task (20 trials)	Cycling during 90% $\dot{\text{V}}\,{\text{O}}_{2}$_*max*_ and Volitional exhaustion	Until exhaustion	During/immediately after	-	RT (during)≈ RT (after): impairment
Kamijo et al. (2004a; 2004b)	*N* = 12 (0) Young	Executive function	S1-S2 RT (Go/No-Go) task (60 trials, 10 min)	Cycling Volitional exhaustion	Until exhaustion	Immediately after (< 3 min)	EEG (CNV, P300)	EMG-RT≈
McMorris et al. (2005)	*N* = 12 (0) Young	Psychomotor task	Whole body choice RT (9 trials)	Cycling 100% maximal power output	Until exhaustion	Immediately after (20 s later)	Blood lactate	RT: impairment
Winter et al. (2007)	*N* = 27 (0) Young	Memory task	Vocabulary learning task (600 training trials + retention)	Running (two sprints, started at 8 km/h, increased every 10 s by 2 km/h) Volitional exhaustion	Until exhaustion	15 min after	Blood catecholamine, BDNF	Learning speed: improvement RT: improvement (1 week later)
Coco et al. (2009)	*N* = 17 (0) Young	Psychomotor task Attentional task	RT task Attention and concentration task	Cycling Volitional exhaustion Lactate infusion (N = 6)	Until exhaustion	5 min after	Blood lactate	RT: impairment Accuracy: impairment
Luft et al. (2009)	*N* = 30 (7) Young	Psychomotor task Executive function Memory task Attentional task	Simple RT (35 correct trials, 90 s) Choice RT (30 correct trials, 90 s) Working memory task (one back task, 30 correct trials, 90 s) Short-term memory task (42 trials, 2–3 min) Continuous monitoring task (30 correct trials, 90 s)	Running Volitional exhaustion	Until exhaustion	10–15 min after	HR variability	Working memory: improvement Others≈
Thomson et al. (2009)	*N* = 163 (0) Young	Psychomotor task	Speed discrimination (decision-making)	Running Volitional exhaustion	Until exhaustion	1 min after	-	Time: improvement Accuracy: impairment
Griffin et al. (2011)	*N* = 47 (0) Young	Memory task Executive function	Face-name matching task Stroop task	Running Volitional exhaustion	Until exhaustion	< 30 min After	Blood BDNF, IGF-1	Face-name matching task: improvement Stroop task≈
Etnier et al. (2016)	*N* = 16 (7) Young	Memory task	Rey Auditory Verbal Learning Test (15 words × 2)	Running Volitional exhaustion	Until exhaustion	Immediately after (after blood sampling)	Blood BDNF	Memory performance: improvement (24 h later)
Hwang et al. (2016)	*N* = 58 (32) Young	Executive function	Stroop test (100 items × 3 conditions, 4 min) Trail making test (< 2 min)	Running Target HR corresponding to 85–90% $\dot{\text{V}}\,{\text{O}}_{2}$_*max*_	10 min	10 min after	Blood BDNF	Stroop test: improvement Trail making test: improvement
Chang et al. (2017)	*N* = 36 (36) Young	Executive function	Stroop test (neutral 60 s, incongruent 60 s)	Running 80% HRR	30 min	15 min after	Cerebral oxygenation	RT≈
Sudo et al. (2017)	*N* = 32 (0) Young	Executive function	Spatial delayed response task (20 trials) Go/No-Go task (20 trials)	Cycling Volitional exhaustion	Until exhaustion	2 min after	Cerebral oxygenation, Blood catecholamine, BDNF, IGF-1, lactate	RT≈ Accuracy≈
Zimmer et al. (2017)	*N* = 119 (41) Young	Executive function	Tower of London	Cycling Volitional exhaustion	Until exhaustion	Immediately after (< 3 min)	Blood lactate	Thinking time: impairment
Du Rietz et al. (2019)	*N* = 29 (0) Young	Executive function Psychomotor task	Cued continuous performance task (modified Go/No-Go task, 80 trials, 11 min) Flanker task (400 trials, 13 min) Choice RT task (72 trials, 10 min)	Cycling 20% delta (difference between gas exchange threshold and $\dot{\text{V}}\,{\text{O}}_{2}$_*peak*_)	20 min	30 min after	EEG	RT≈ Accuracy≈
Hill et al. (2019)	*N* = 13 (0) Young	Executive function	Flanker task (100 trials, < 3 min)	Cycling and arm cranking Volitional exhaustion	Until exhaustion	Immediately after	-	Cycling: impairment Arm cranking: improvement
Coco et al. (2020a)	*N* = 30 (?) Young = 15 Old = 15	Psychomotor task Executive function	Simple RT Stroop color word test (50 names, 50 circles, and 50 words) Trail making test	Cycling Volitional exhaustion	Until exhaustion	Immediately after	Blood lactate	Simple RT: impairment Stroop Color Word Test: impairment Trail Making Test: improvement (Young)
Loprinzi et al. (2021)	*N* = 120 (77) Young	Memory task	Word list memory task (15 words)	Running 75% HRR	20 min	5 min after	-	Memory: improvement
Marin Bosch et al. (2021)	*N* = 18 (0) Young	Psychomotor task Memory task	Psychomotor vigilance task (RT) Associative memory task (8 series of 6 successive pictures)	Cycling 75% maximal cardiac frequency	15 min	24 min after (Psychomotor task) and 69 min after (memory task)	Neural activity (fMRI), Blood endocannabinoids, BDNF	RT≈ Accuracy≈

F, females; N, number of participants; RT, reaction time; W, watts; $\dot{\text{V}}\,{\text{O}}_{2}$*_peak_*, peak oxygen uptake; EMG, electromyogram; LT, lactate threshold; HR, heart rate; HRR, heart rate reserve; AT, anaerobic threshold; VT, ventilatory threshold; EEG, electroencephalogram; fNIRS, functional near-infrared spectroscopy; CNV, contingent negative variation; P300, positive 300; $\dot{\text{V}}\,{\text{O}}_{2}$*_max_*, maximal oxygen uptake; BDNF, brain-derived neurotrophic factor; IGF-1, insulin-like growth hormone factor-1; fMRI, functional magnetic resonance imaging. ≈, no effect.

Conversely, cognitive performance after high-intensity exercise is heterogeneous; with improvements (Winter et al., 2007; Luft et al., 2009; Thomson et al., 2009; Griffin et al., 2011; Etnier et al., 2016; Hwang et al., 2016; Hill et al., 2019; Coco et al., 2020a; Loprinzi et al., 2021), impairments (Fery et al., 1997; McMorris et al., 2005; Coco et al., 2009, 2020a; Thomson et al., 2009; Zimmer et al., 2017; Hill et al., 2019), and no changes (Travlos and Marisi, 1995; Brisswalter et al., 1997; Kamijo et al., 2004a,b; Luft et al., 2009; Griffin et al., 2011; Chang et al., 2017; Sudo et al., 2017; Du Rietz et al., 2019; Marin Bosch et al., 2021) reported within the literature. These findings suggest that cognitive performance after high-intensity exercise appears to be dependent on experimental design (*see below*). In the following sections, we discuss the methodological, physiological, and psychological factors that affect cognitive performance “*during*” and “*after*” high-intensity exercise.

## Methodological factors

Here we discuss the potential methodological and experimental factors that contribute to the inconsistent findings. These include the following: timing of cognitive task, type of cognitive task, physical fitness, exercise mode/duration, and age.

### Timing of cognitive task

When participants perform cognitive tasks during exercise, they perform the exercise and cognitive tasks simultaneously (i.e., a dual-task paradigm). However, when cognitive tasks are performed after exercise, participants only perform a single task. A meta-analysis reported higher effect sizes in single-task conditions (after exercise) when compared with dual-task conditions (during exercise) (Lambourne and Tomporowski, 2010), while another meta-analysis reported that effect sizes were not different between single and dual-task conditions (Chang et al., 2012). Furthermore, McMorris and Hale (2012) undertook statistical analyses and found that there were no differences in effect sizes obtained during compared after exercise. Nevertheless, as recently highlighted (McMorris, 2021), the timing of the cognitive tasks is typically less considered within the literature.

Table 1 indicates that the adverse effects are most prominent *during* high-intensity exercise. These findings are corroborated by a recent review and suggest that impairments in cognitive performance are more likely to occur during high-intensity exercise (Zheng et al., 2021). Based on the assumption that metabolic resources are limited in the brain, extensive activation in several brain regions (e.g., motor and sensory cortices) may attenuate higher-order functions of the PFC and impair cognitive performance (Dietrich and Audiffren, 2011). Furthermore, in the majority of the included studies, cognitive performance was assessed using manual responses where activations of the motor-related areas are required. Given a limited capacity of the brain to simultaneously activate multiple regions involved in cognitive performance and high-intensity exercise, it is plausible that cognitive performance is more likely to be impaired during high-intensity exercise, particularly when both cognitive and physiological demands are high. Indeed, in four studies reporting cognitive improvement during high-intensity exercise (Huertas et al., 2011; Shields et al., 2011; Davranche et al., 2015; Tempest et al., 2017), exercise intensities were relatively less demanding (i.e., HR < 170 bpm). Relatively lower physiological demands may be responsible for cognitive improvements during high-intensity exercise in these studies. Furthermore, McMorris (2021) argued that performance would depend to a large extent on the perception of task costs (demands) and resources available. These two judgments may be difficult to make in the dual-task situation, leading to over-or under-confidence. This could alter motivation, which would affect cognitive performance.

A recent meta-analysis demonstrated that acute high-intensity exercise had a small, significant facilitating effect on cognitive performance *after* high-intensity exercise (Moreau and Chou, 2019). In the current review, we observed that cognitive performance after high-intensity exercise is inconsistent: and improvements, impairments, and no changes were reported. EEG studies reported reductions in P3 amplitudes after high intensity (Kamijo et al., 2004b) or HIIE (Kao et al., 2017). On the contrary, Du Rietz and colleagues reported improvements in P3 amplitude and delta power reflecting executive and sustained attention after high-intensity exercise (Du Rietz et al., 2019). These findings suggest that brain activity after exercise may be dependent on the experimental design employed (e.g., exercise intensity, time delay after exercise). Indeed, most physiological changes start to recover immediately after high-intensity exercise (Ide et al., 2000; Gonzalez-Alonso et al., 2004; Curtelin et al., 2017; Sudo et al., 2017). Thus, rapid recovery of physiological variables to homeostatic resting levels may, at least in part, explain the contradictory findings related to cognitive performance after high-intensity exercise. To explore this possibility, we summarized the impacts of the timeframe in which cognitive performance was assessed after exercise (Table 2). When multiple cognitive tasks were examined in a single study, or when both improvement and impairment were reported in a cognitive task (e.g., improvement in RT and impairment in accuracy), all results are reported. We observed heterogeneous findings when cognitive performance was assessed within 5 min of completing high-intensity exercise. Intriguingly, however, no impairments were reported when the timing of cognitive tasks was > 6 min after exercise. These findings support the notion that a rapid recovery to homeostatic resting levels is critical for cognitive performance after high-intensity exercise. Taken collectively, we propose that the effects of high-intensity exercise on cognitive performance are closely related to, and impacted by, the timing of cognitive task (during vs. after exercise, and the time delay after exercise).

**TABLE 2:** Summary of impacts of time delay after exercise.

0–5 min			6–10 min			11–20 min			>20 min		
Improvement (↑)	No change (↔)	Impairment (↓)	↑	↔	↓	↑	↔	↓	↑	↔	↓
⬤⬤⬤⬤⬤	⬤⬤⬤⬤⬤	⬤⬤⬤⬤⬤⬤⬤⬤⬤	⬤⬤			⬤⬤	⬤⬤⬤⬤⬤		⬤	⬤⬤⬤⬤⬤⬤	

Number of black circles indicates the number of studies.

### Type of cognitive task

Different brain regions are thought to be activated during different cognitive tasks (Macintosh et al., 2014; Chen A. G. et al., 2016; Won et al., 2019). Thus, we can assume that the type of cognitive task is one of the factors that determine how acute high-intensity exercise impacts cognitive performance. This may be particularly relevant when exercise and cognitive tasks are concurrently performed since multiple brain regions are presumably activated. In this review, we classified the type of cognitive task into executive function, psychomotor, memory, and attentional tasks (Table 3). Executive function encompasses several subdomains and consists of basic components of inhibition, working memory, and cognitive flexibility (Diamond, 2013). Thus, based on the included studies, we classified executive function into response inhibition (Go/No-Go task), interference control (Flanker task, Simon task, and Stroop task), working memory (n-back task and spatial delayed response task), and others (soccer-specific task, Wisconsin card sorting task, Cedar Operator Workload Assessment Tool, Tower of London, and Trail making test).

**TABLE 3:** Summary of impacts of the type of cognitive task.

	Executive function			Psychomotor task			Memory task			Attentional task		
	Improvement (↑)	No change (↔)	Impairment (↓)	↑	↔	↓	↑	↔	↓	↑	↔	↓
During	***⬤	†*#	†**$$##⬤⬤			⬤⬤⬤⬤		⬤⬤		⬤⬤	⬤⬤	⬤
After	*$#⬤⬤	†††*$$#	*$⬤	⬤	⬤⬤⬤⬤⬤⬤	⬤⬤⬤⬤	⬤⬤⬤⬤	⬤⬤	⬤		⬤	⬤

Number of symbols indicates the number of studies.

^†^Response inhibition (Go/No-Go task).

*Interference control (Flanker task, Simon task).

^$^Interference control (Stroop task).

^#^Working memory (n-back task, spatial delayed response task).

⬤ Others (soccer-specific task, Wisconsin card sorting task, Cedar Operator Workload Assessment Tool, Tower of London, and Trail making test), psychomotor task, memory task, attentional task.

During high-intensity exercise, impairments in cognitive performance were prominent in executive function and psychomotor tasks (Table 3), which is thought to be closely related to dual-task paradigm. Thus, in most cognitive tasks, adverse effects are more likely to occur during high-intensity exercise. Conversely, we found improvements in several studies. In three studies (Huertas et al., 2011; Davranche et al., 2015; Tempest et al., 2017), cognitive improvements were observed in interference control (i.e., Flanker task and Simon task). These findings imply that performances in these cognitive tasks may benefit from high-intensity exercise relative to the other subcomponents of executive function. Furthermore, in the study reporting improvements in soccer-specific cognitive tasks (both soccer-specific and attentional tasks) in college soccer players (McMorris and Graydon, 1997), the cognitive tasks were probably autonomous to the soccer players and therefore less demanding. Hence, cognitive improvements during high-intensity exercise appear to be related to cognitive demands.

A meta-analysis review reported that facilitating effects of performance were similarly observed in subcomponents of executive function (i.e., working memory, inhibitory control, cognitive flexibility, and attention) after high-intensity exercise (Moreau and Chou, 2019). In the present review, we failed to find clear associations between the type of cognitive task and cognitive performance after high-intensity exercise. It should be noted, however, that improvements in memory performance were often observed after high-intensity exercise (Winter et al., 2007; Griffin et al., 2011; Etnier et al., 2016; Loprinzi et al., 2021). In particular, high-intensity exercise may be beneficial for retention (Winter et al., 2007; Etnier et al., 2016; Loprinzi et al., 2021). Additional studies are necessary to further elucidate potential physiological mechanisms underlying the improvement. In this review, we may not have been sufficiently powered to identify the effects of different cognitive tasks/domains. Further research is required to establish the effects of high-intensity exercise on a variety of cognitive tasks and domains.

### Physical fitness level of participants

It has been previously speculated that exercise-cognition interaction is influenced by physical fitness (Lambourne and Tomporowski, 2010; Chang et al., 2012). For example, despite matched relative exercise intensity, individuals with lower physical fitness levels were more susceptible to cognitive impairments during high-intensity exercise, when compared with those who had higher aerobic capacities (Brisswalter et al., 1997; Labelle et al., 2013). Furthermore, choice RT performance gradually improves during incremental exercise until at ∼75% $\dot{\text{V}}\,{\text{O}}_{2}$_*max*_ in young soccer players (Chmura et al., 1994). Aerobic capacity has been suggested to be one of the moderators that affect cognitive performance in response to high-intensity exercise (Browne et al., 2017). McMorris (2021) claimed that fitness levels would affect the individual’s perception of effort costs and, hence, their motivation level. Further, well-powered studies are also necessary to clarify the relationship between cognitive performance and physical fitness before this theory can be confirmed.

### Exercise mode

A previous meta-analysis suggested that exercise mode is one of the factors that affect exercise-cognition interaction (Lambourne and Tomporowski, 2010). We summarized the impacts of high-intensity cycling and running in Table 4. The effects of high-intensity exercise on cognitive performance during cycling were inconsistent, but reductions in cognitive performance appeared to be more likely during cycling. Running is kinematically less stable compared to cycling, and thus it may be difficult to complete cognitive performance during high-intensity running. Indeed, only two studies examined cognitive performance during high-intensity running, and both reported impairments in cognitive performance (Smith et al., 2016; Stone et al., 2020). Given the nature of the dual-task paradigm, running may be more detrimental to cognitive performance relative to cycling during high-intensity exercise.

**TABLE 4:** Summary of impacts of exercise mode.

	Cycling (+ arm cranking)			Running		
	Improvement (↑)	No change (↔)	Impairment (↓)	↑	↔	↓
During	⬤⬤⬤⬤⬤⬤	⬤⬤⬤⬤⬤⬤⬤	⬤⬤⬤⬤⬤⬤⬤⬤⬤⬤⬤⬤			⬤⬤
After	⬤⬤	⬤⬤⬤⬤⬤⬤⬤⬤⬤⬤	⬤⬤⬤⬤⬤⬤⬤⬤	⬤⬤⬤⬤⬤⬤⬤⬤	⬤⬤⬤⬤	⬤

Number of black circles indicates the number of studies.

After high-intensity cycling, in most cases, we observed no changes or impairments in cognitive performance. Conversely, after high-intensity running, improvements or no changes in cognitive performance were predominant. Since participants perform only a cognitive task after exercise (i.e., single task) regardless of exercise mode, the findings are somewhat unexpected. There are several physiological differences in ventilatory and metabolic responses, HR, and motor unit recruitment between cycling and running (Millet et al., 2009). Hence, the recovery of physiological variables to resting levels could be different between high-intensity cycling and running. Collectively, these findings suggest that the mode of exercise is one of the factors that affect cognitive performance, and these effects are presumably influenced by the timing of cognitive task (during vs. after exercise, and the time delay after exercise). Future studies are required to understand how exercise mode influences cognitive performance after high-intensity exercise.

### Exercise duration

Exercise duration also potentially affects cognitive performance in response to high-intensity exercise. We summarized the relationship between exercise duration and cognitive performance in Table 5. During exercise, cognitive impairments were observed when exercise duration was < 10 min. The findings were inconsistent when exercise duration was > 11 min or exhaustive exercise. Thus, it is less likely that exercise duration *per se* affects cognitive performance. Rather, interactions between exercise duration and intensity would be more important for cognitive performance during high-intensity exercise. In most studies that assessed cognitive performance after high-intensity exercise, exercise was continued until exhaustion (Table 5). Nevertheless, the results were heterogeneous and suggest that exercise duration in isolation is not critical for cognitive performance after exhaustive exercise. Assuming that the duration of high-intensity exercise is likely limited, exercise duration alone is, therefore, not likely to determine cognitive performance.

**TABLE 5:** Summary of impacts of exercise duration.

	<10 min			11–20 min			>20 min			Until exhaustion		
	Improvement (↑)	No change (↔)	Impairment (↓)	↑	↔	↓	↑	↔	↓	↑	↔	↓
During		⬤⬤	⬤⬤⬤⬤⬤⬤⬤	⬤	⬤⬤		⬤⬤	⬤	⬤⬤⬤		⬤⬤	⬤⬤⬤
After	⬤⬤	⬤		⬤	⬤⬤⬤⬤⬤			⬤		⬤⬤⬤⬤⬤⬤⬤	⬤⬤⬤⬤⬤⬤⬤⬤⬤	⬤⬤⬤⬤⬤⬤⬤⬤⬤

Number of black circles indicates the number of studies.

### Age

Coco and colleagues compared the effects of exhaustive exercise on the cognitive performance of young and older adults (Coco et al., 2020a). They reported impairments in the performance of simple RT and the Stroop color-word test after exhaustive exercise in both groups. However, improvements in trail making test were observed only in the younger group. These results suggest that the effects of high-intensity exercise on cognitive performance may be different in young and older adults. Cerebral perfusion appears to be lower in older individuals during high-intensity exercise, although the cerebral extraction of glucose, lactate, and oxygen is similar (Braz and Fisher, 2016). Thus, age may be one factor that may influence cognitive performance and reduced cerebral perfusion could affect cognitive performance in older individuals in particular. Given the brevity of studies in this area, definitive conclusion on the impact of age is not yet feasible.

## Physiological factors

As noted above, high-intensity exercise induces a variety of physiological effects within the human brain. Here, we summarize and discuss the physiological factors that are linked to cognitive performance during and after high-intensity exercise and we identify some of the potential physiological factors that may contribute to the inconsistent findings. These include the separate and combined effects of cerebral blood flow (CBF), cerebral oxygenation, cerebral metabolism, neuromodulation by neurotransmitters and neurotrophic factors, and various psychological factors.

### Cerebral blood flow

During exercise, CBF is regulated by complex interactions between neural activity and metabolism, partial pressure of oxygen, carbon dioxide (CO_2_), blood pressure, cardiac output, and sympathetic nervous system activity (Ogoh and Ainslie, 2009; Smith and Ainslie, 2017). CBF gradually increases during mild- to moderate-intensity exercise in response to neural activity and metabolism (Ogoh and Ainslie, 2009). However, during high-intensity exercise, hyperventilation-induced hypocapnia constricts the cerebral vessels, thereby reducing CBF (Ogoh and Ainslie, 2009; Smith and Ainslie, 2017). This suggests that brain metabolic demands might be inadequate during high-intensity exercise. Ogoh et al. (2014) reported that an increase in CBF, achieved using CO_2_ inhalation, did not affect cognitive performance during prolonged moderate-intensity exercise (Ogoh et al., 2014). More recently, Komiyama and colleagues tested the hypothesis that a reduction in CBF is directly linked to impairment in cognitive performance during high-intensity exercise (Komiyama et al., 2020). By restoring CBF *via* CO_2_ inhalation, the authors demonstrated that middle cerebral artery (MCA) velocity (a surrogate for CBF) did not prevent impaired cognitive performance during high-intensity exercise. These results suggest that a reduction in CBF *per se* may not be responsible for impaired cognitive performance during high-intensity exercise. However, given that CBF supplies oxygen and nutrients, the association between cognitive performance and regional CBF (e.g., blood flow to the PFC) in response to high-intensity exercise should be further investigated. In particular, a recent study indicated physiological “uncoupling” between the PFC oxygenation and MCA velocity during high-intensity exercise with CO_2_ inhalation (Hansen et al., 2020). Follow-up studies are needed to fully understand the association between regional CBF and cognitive performance in response to high-intensity exercise.

### Cerebral oxygenation

Cerebral oxygenation reflects the balance between cerebral oxygen availability and utilization (Boushel et al., 2001; Komiyama et al., 2017), which is generally measured from the PFC. Cerebral oxygenation reduces during high-intensity exercise close to maximal intensity (Rooks et al., 2010). Some studies suggest that a reduction in cerebral oxygenation is not associated with impairments in cognitive performance during high-intensity exercise (Ando et al., 2011; Schmit et al., 2015; Tempest et al., 2017). In contrast, others have indicated that impairments in cognitive performance were accompanied by reduction in cerebral oxygenation during high-intensity exercise (Mekari et al., 2015; Stone et al., 2020). The latter studies suggest that impairments in cognitive performance may be associated with attenuated PFC oxygenation. However, several studies have shown that cognitive performance improved during acute moderate-intensity exercise in hypoxia despite substantial reductions in cerebral oxygenation (Ando et al., 2013; Komiyama et al., 2015, 2017). Hence, it is likely that a reduction in cerebral oxygenation, in isolation, does not result in impaired cognitive performance. However, reduction in cerebral oxygenation during high-intensity exercise may impair cognitive performance in concert with other physiological factors.

Cerebral oxygenation starts to recover immediately after maximal exercise (Gonzalez-Alonso et al., 2004; Sudo et al., 2017). Notably, the degree of recovery of cerebral oxygenation following maximal exercise may be associated with cognitive performance (Sudo et al., 2017). This finding suggests that the recovery of cerebral oxygenation after high-intensity exercise may, at least in part, account for the differential effects of high-intensity exercise on cognitive performance between single (i.e., after) and dual (i.e., during) conditions.

### Cerebral metabolism

It is generally accepted that blood glucose is the primary energy source for the brain at rest (Gold, 1995). Komiyama et al. (2016) reported that cognitive performance improves during moderate-intensity exercise after skipping breakfast. This suggests that substrates other than glucose may compensate for the reduced availability of blood glucose during moderate-intensity exercise. It is plausible that the same would be true for high-intensity exercise. Indeed, blood glucose uptake is thought to be reduced in the brain during high-intensity exercise (Kemppainen et al., 2005). In contrast, blood lactate substantially increases during/after high-intensity exercise, and it is taken up by the brain (Ide et al., 2000; Gonzalez-Alonso et al., 2004; Quistorff et al., 2008; Siebenmann et al., 2021). Several studies suggested that blood lactate would provide energy that contributes to improvements in cognitive performance following HIIE (Tsukamoto et al., 2016; Hashimoto et al., 2018; Herold et al., 2022). In particular, Hashimoto et al. (2018) directly measured lactate uptake in the brain after HIIE and suggested that lactate production in extra-cerebral tissues supports brain function. On the contrary, Coco et al. (2020b) suggested that high levels of blood lactate have detrimental effects on cognitive performance. Interestingly, Coco et al. (2009) indicated that intravenous lactate infusion of a lactate solution impaired attentional performance. Hence, further studies are warranted to investigate how blood lactate acts as a mediator of exercise-induced alterations in cognitive performance (Ando et al., 2022).

### Neuromodulation by neurotransmitters and neurotrophic factors

In humans, it is less clear how acute exercise alters central neurotransmitter release due to technical and methodological challenges. Nevertheless, given that rodent studies indicate that acute exercise releases neurotransmitters in the brain (Meeusen et al., 2001; Hasegawa et al., 2011; Goekint et al., 2012; Chen C. et al., 2016), acute exercise is likely to influence brain circuits involving a number of neurotransmitters including dopamine and noradrenaline (McMorris, 2016a; Ando et al., 2020). Dopamine and noradrenaline modulate the strength of the PFC network connections, and regulation of dopamine and noradrenaline is required for appropriate prefrontal cognitive function (Arnsten, 2011). Furthermore, excess noradrenaline and dopamine appear to weaken the signal-to-noise ratio, which may result in impairments in the PFC function (Arnsten, 2011; Cools and Arnsten, 2021). Hence, the available literature suggests that excess neuromodulators in the brain may have adverse effects on cognitive performance during/after high-intensity exercise. High-intensity exercise also seems to increase brain-derived neurotrophic factors (BDNFs) (Ferris et al., 2007; Winter et al., 2007; Fernandez-Rodriguez et al., 2021) and insulin-like growth hormone factor-1 (IGF-1) (Sudo et al., 2017). Several studies have implicated that changes in BDNF are associated with cognitive improvement induced by acute exercise (Winter et al., 2007; Lee et al., 2014; Skriver et al., 2014; Hwang et al., 2016). However, BDNF and IGF-1 are known to play a crucial role in angiogenesis, synaptogenesis, and neurogenesis following long-term exercise (Cotman and Berchtold, 2002; Voss et al., 2011; Nieto-Estevez et al., 2016). High concentrations of dopamine, noradrenaline, and BDNF are necessary for long-term potentiation, which is essential for long-term memory (McMorris, 2021). A couple of studies have shown positive effects of high-intensity exercise on long-term memory (Winter et al., 2007; Griffin et al., 2011). At present, it is premature to conclude that changes in BDNF and IGF-1 play a role in cognitive performance during/after high-intensity exercise.

## Psychological factors

In most studies, psychological factors are typically not well considered when attempting to elucidate the effects of high intensity on cognitive performance. However, as suggested by McMorris (2021), psychological factors such as the motivation and perception of effort may affect the acute exercise–catecholamine–cognition interaction. Cantelon and Giles also suggested that psychological factors are moderating factors that affect exercise–cognition interaction (Cantelon and Giles, 2021). At present, and given the lack of empirical evidence, further investigations are needed to investigate the association between psychological factors and cognitive performance during and after high-intensity exercise.

## Integration of physiological and psychological factors

**FIGURE 1:**
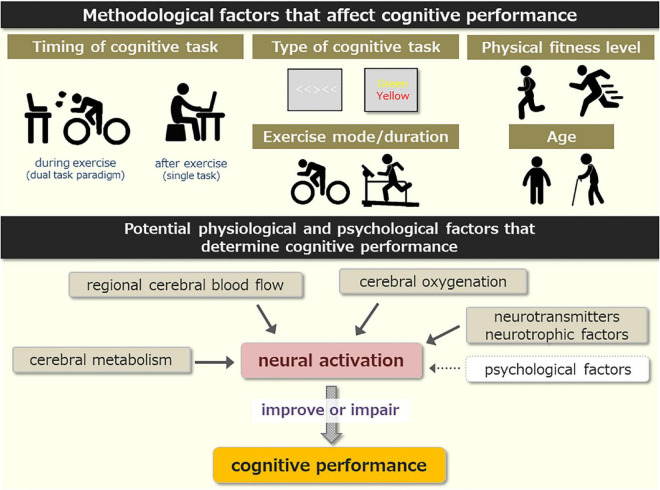
(Upper) Summary of methodological factors that affect cognitive performance in response to high-intensity exercise. (Lower) Potential physiological and psychological factors that mediate cognitive performance.

It is likely that the effects of high-intensity exercise on cognitive performance are multifactorial and determined by the integration of several physiological and psychological factors (Figure 1). We propose that interactions of these factors influence neural activity associated with cognitive performance and that this determines cognitive performance during and after high-intensity exercise. This is consistent with McMorris (2021) claiming that the perception of physiological stress affects the motivation and perception of effort costs. However, the current literature base is insufficient to substantiate this speculation and this should be the focus of future research in this area. A recent fNIRS study detected cognitive task-related hemodynamic changes from the left PFC during high-intensity exercise (Tempest and Reiss, 2019). Furthermore, an fMRI study indicated that HIIE decreased brain activation associated with cognitive performance (Mehren et al., 2019). Thus, future studies using sophisticated neuroimaging methods (e.g., fNIRS, fMRI, and PET) are required to fully understand how a single bout of high-intensity exercise affects cognitive performance.

## Conclusion

This narrative review summarized the literature examining the effects of acute high-intensity exercise on cognitive performance. We propose that the effects of high-intensity exercise on cognitive performance are primarily affected by a variety of methodological, physiological, and psychological factors. Specifically, these include the timing of cognitive task (during vs. after exercise, and the time delay after exercise), cognitive task(s), fitness level, exercise mode/duration, and age. It is also likely that a complex interaction between changes in regional CBF, cerebral oxygenation, cerebral metabolism, neurotransmitters/neurotrophic factors, and a variety of psychological factors contributes to the heterogeneous findings reported. The review is likely to have implications for recreational, sporting, and occupational activities where high cognitive and physiological demands are required simultaneously.

## Author contributions

MS, JC, TM, and SA drafted the manuscript. All authors approved the final version of the manuscript.
